# Green Surgery Awareness and Challenges: A Survey Among Members of the Japan Society for Endoscopic Surgery

**DOI:** 10.1111/ases.70087

**Published:** 2025-05-29

**Authors:** Naoki Ikenaga, Masafumi Nakamura, Mizuki Hirasawa, Takeshi Naitoh, Yoshiharu Sakai, Tomonori Habuchi, Yuko Kitagawa

**Affiliations:** ^1^ Department of Surgery and Oncology Graduate School of Medical Sciences, Kyushu University Fukuoka Japan; ^2^ Secretariat of the Japan Society for Endoscopic Surgery Tokyo Japan; ^3^ Department of Lower Gastrointestinal Surgery Kitasato University School of Medicine Sagamihara Japan; ^4^ Department of Surgery Osaka Red‐Cross Hospital Osaka Japan; ^5^ Department of Urology Akita University Graduate School of Medicine Akita Japan; ^6^ Department of Surgery Keio University School of Medicine Tokyo Japan

**Keywords:** endoscopic surgery, green surgery, minimally invasive surgery, survey, sustainability

## Abstract

**Introduction:**

Global warming poses an urgent challenge, with the healthcare sector being a significant contributor to greenhouse gas (GHG) emissions. The rise in minimally invasive surgery, which often depends on energy‐intensive technologies and single‐use devices, further exacerbates this environmental burden. The concept of “Green Surgery” aims to reduce the environmental footprint while maintaining patient care standards. Yet awareness and attitudes of surgeons in Japan regarding these practices are largely unknown.

**Methods:**

The Japanese Society for Endoscopic Surgery (JSES)'s Working Group on Environmental Issues conducted an online survey with 21 questions exploring knowledge of Green Surgery and perspectives on sustainable practices. The survey was distributed to all JSES members, with anonymous responses collected between October 18 and November 12, 2024.

**Results:**

A total of 1601 participants (10.1%) responded. Awareness of Green Surgery was limited, with only 5% demonstrating a well‐developed understanding, though 67% expressed concerns about GHG emissions and operating room waste. Environmentally friendly practices were reported by only 14% of respondents' facilities, with perceived cost increases (55%) and workflow changes (39%) as key barriers. Nonetheless, 82% were willing to adopt sustainable practices, preferring reusable instruments (74%) and remanufactured single‐use devices (66%).

**Conclusion:**

This survey highlights the challenges and opportunities in promoting Green Surgery in Japan. Despite limited awareness, there is considerable interest in adopting sustainable practices. These findings support the JSES in spearheading initiatives to integrate sustainability into surgical practices, thereby aiding in achieving global climate goals.

## 1 Introduction

The escalating threat of global warming is one of the most critical challenges of the 21st century, necessitating urgent and coordinated global action [1, 2]. The Paris Agreement, adopted at COP21 in 2015, established a common goal among 195 countries to limit global warming to well below 2°C, with an ambitious target of keeping it close to 1.5°C above pre‐industrial levels. To achieve this, Japan aims to reduce its greenhouse gas (GHG) emissions by 46% by fiscal year 2030 compared to fiscal year 2013, aligning with its long‐term commitment to reach net‐zero emissions by 2050 [3].

The healthcare sector is a significant contributor to the global climate crisis, with its carbon footprint steadily increasing in Japan, which accounted for 5.2% of the nation's total emissions (72.0 MtCO_2_e) in 2015 [4]. In the United States, the healthcare sector is responsible for approximately 10% of national emissions [5], highlights the urgent need for sustainable practices in this field. Operating rooms (ORs) are particularly large contributors to GHG emissions due to high energy consumption, extensive use of disposable instruments, and substantial waste generation. The environmental impact has intensified with the increasing adoption of minimally invasive surgery, which, despite delivering benefits for patient outcomes, often relies on energy‐intensive technologies and single‐use devices.

In response to the environmental challenges posed by healthcare, the concept of “Green Surgery” has emerged, advocating practices that minimize carbon footprints while maintaining or enhancing patient outcomes [6]. International organizations, including the International Federation of Societies of Endoscopic Surgeons (IFSES), have initiated efforts to assess the environmental impact of surgical practices, reduce emissions, and educate healthcare professionals on sustainability.

As a member of IFSES, the Japanese Society for Endoscopic Surgery (JSES) has established a Working Group on Environmental Issues [7] to confront these challenges. However, awareness of environmentally friendly surgical practices remains low in Japan, highlighting the need for enhanced promotion of sustainable practices.

A survey was conducted to assess the awareness, attitudes, and practices related to Green surgery among JSES members to address the pressing environmental impact of surgical practices. This survey aimed to gather foundational data on sustainable surgical practices in Japan and identify barriers and opportunities that will inform the JSES's future initiatives and strategies following global sustainability goals.

## 2 Materials and Methods

The survey was designed by the Working Group on Environmental Issues of the Future Vision Committee of the JSES. The survey instrument consisted of three sections: 1. demographic and professional information; 2. current understanding and awareness of Green Surgery; and 3. perspectives on future initiatives. The instrument included 21 questions formatted as multiple‐choice, Likert scale, and open‐ended responses (File S1).

The survey was created using Microsoft Forms, and an e‐mail invitation with a link was sent to all JSES members. Responses were collected anonymously between October 18 and November 12, 2024. Participation was voluntary, ensuring confidentiality, and participants were informed that the results would be utilized solely for academic purposes and publicly shared. The survey did not include questions that could cause psychological distress, discomfort, or probe into sensitive personal experiences. Therefore, ethical approval and informed consent were considered not to be required. The responses were aggregated and analyzed using descriptive statistics.

## 3 Results

A total of 1601 (10.1%) participated in the survey, drawn from 15,857 members. The largest groups of respondents were in their 40s (35%) and 50s (38%). Most respondents were male (91%), followed by females (9%). Most respondents had 20–29 (41%) or 10–19 (25%) years of medical experience. Staff physicians accounted for 45% of responses, followed by department heads at 33% and residents at 3%. Participants primarily specialized in gastrointestinal and general surgery (69%), followed by thoracic surgery (10%), urology (8%), gynecology (7%), pediatric surgery (4%), and orthopedic surgery (0.4%). Detailed participant demographics are shown in Table S1.

### 3.1 Awareness and Knowledge of Green Surgery

**FIGURE 1:**
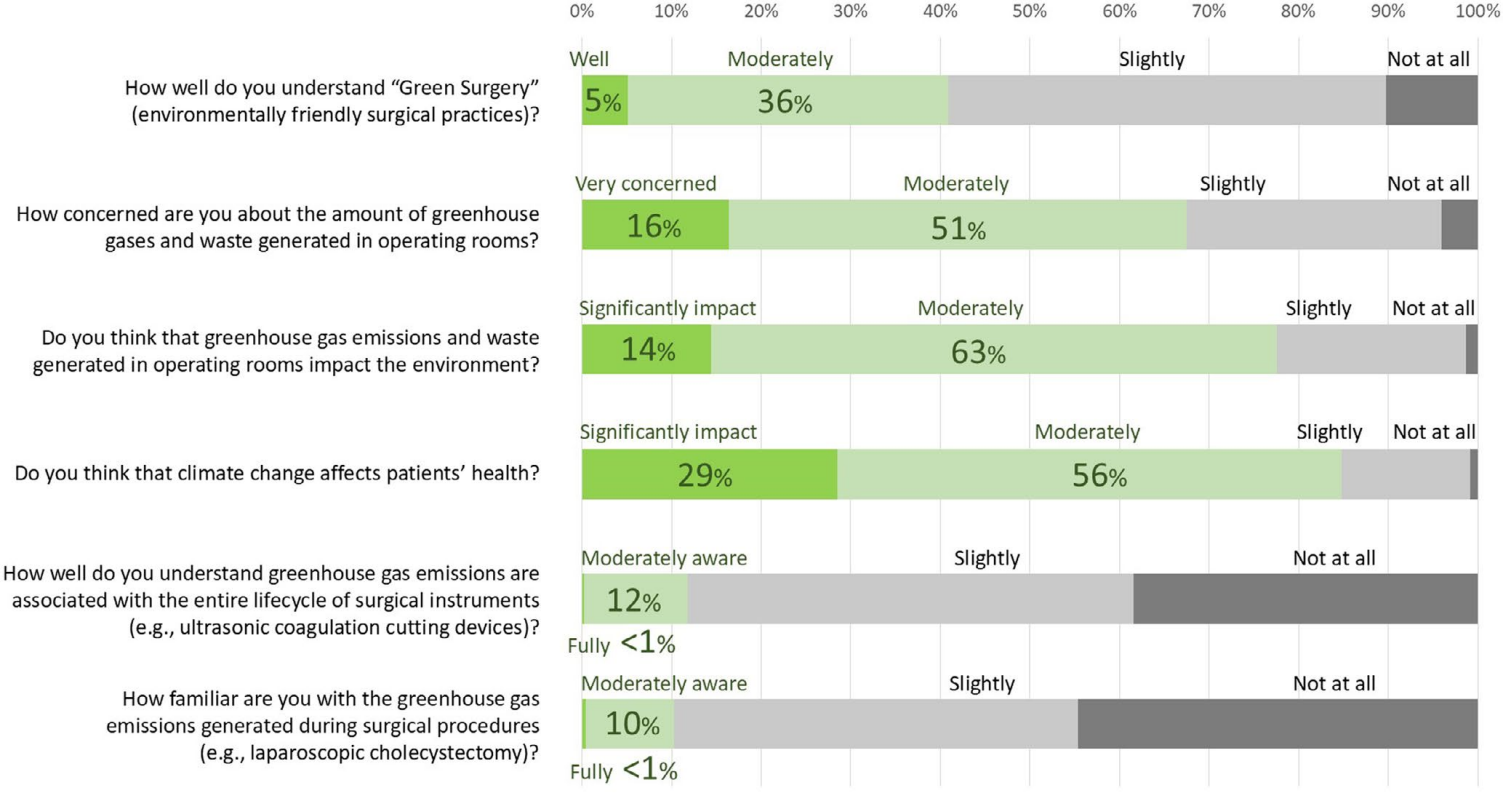
Current awareness and knowledge of Green Surgery among participants. GHG, a greenhouse gas; ORs, operating rooms.

Figure 1 summarizes participants' awareness and knowledge regarding environmentally sustainable surgical practices and their environmental impact. Awareness of “Green Surgery” [6] was generally low, with only 5% of respondents indicating a well‐developed understanding, while 36% reported a moderate understanding. In contrast, many participants expressed substantial concern about the environmental impact of ORs; 16% were very concerned, and 51% had moderate concerns regarding GHG emissions and waste generated in ORs. Additionally, 63% perceived GHG emissions and waste as having a moderate environmental impact, whereas 14% considered the impact significant. Regarding health‐related perceptions, 56% of respondents believed climate change moderately affects patient health, while 29% felt the impact was significant. However, knowledge about specific GHG emissions linked to surgical instruments and procedures was limited, as most participants (88% for surgical instruments and 90% for surgical procedures) reported being slightly aware or unaware of these emissions. These findings highlight a significant knowledge gap concerning the environmental impact of surgical practices and emphasize the need for increased awareness and education, particularly regarding sustainable surgical practices.

### 3.2 Current Engagement in Green Operating Room Management

**FIGURE 2:**
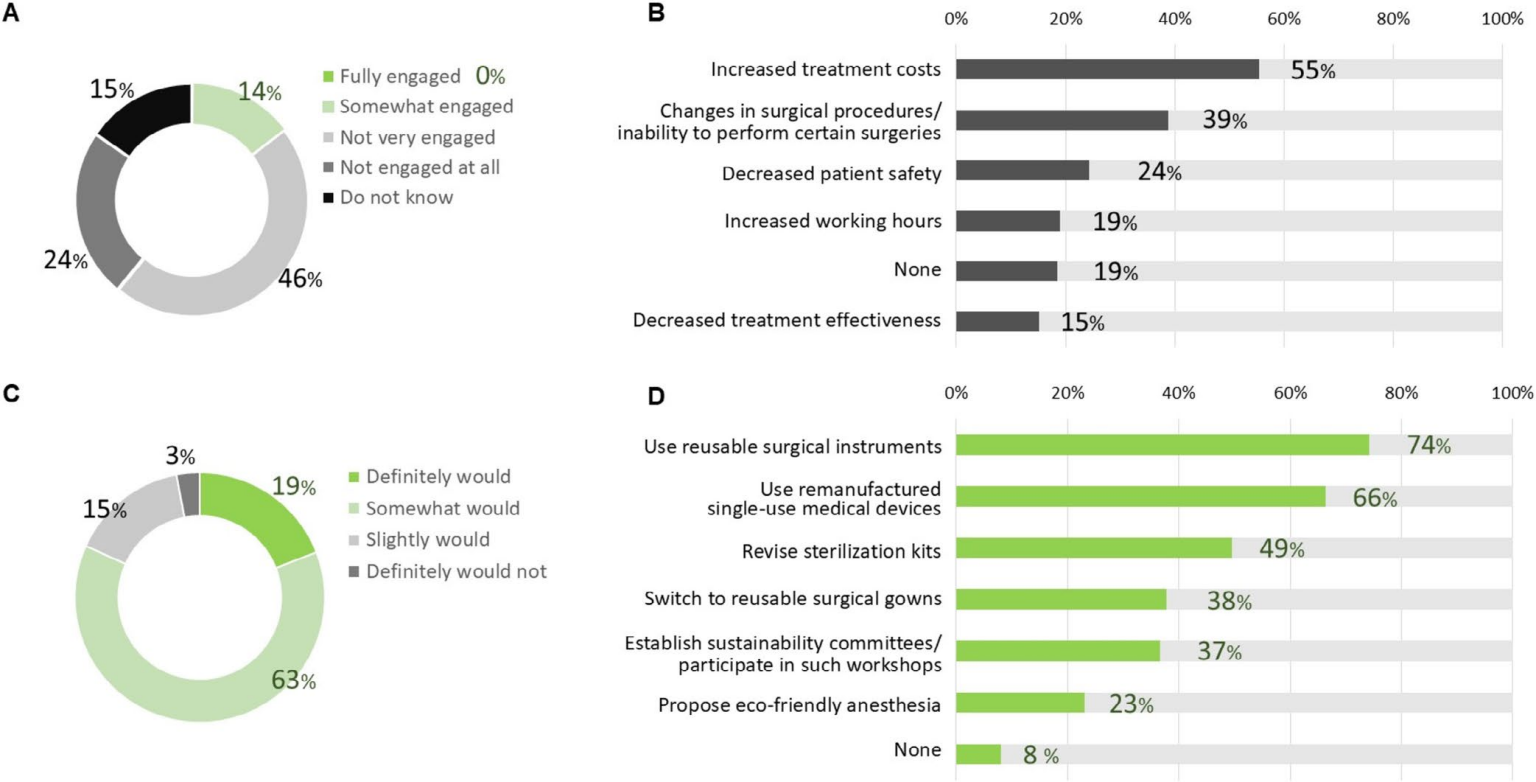
Barriers and perspectives on future initiatives in Green Surgery. (A) Proportion of institutions engaged in environmentally friendly operating room management. Responses to the question: “Is your facility actively engaged in environmentally friendly operating room management?” (B) Concerns considered by participants to promote Green Surgery. (C) Proportion of participants who wish to engage in Green Surgery. Responses to the question: “Would you like to engage in Green Surgery practices?” (D) Actions preferred by participants for the implementation of Green Surgery in the future.

Figure 2A,B illustrate the current engagement levels in environmentally friendly OR management and the perceived barriers to implementing Green Surgery practices. Most respondents reported low engagement in sustainable practices at their facilities. None indicated that their facility was “fully engaged,” while 46% classified their facilities as “not very engaged,” and 24% stated that they were “not engaged at all.”

Furthermore, 15% were unsure about their facility's level of involvement, reflecting a lack of awareness or communication regarding green initiatives (Figure 2A). The most frequently cited barrier to adopting Green Surgery practices was the perception of increased treatment costs, reported by 55% of respondents. Other notable barriers included changes to surgical procedures (39%), concerns about patient safety (24%), and increased working hours (19%). Interestingly, only 15% cited decreased treatment effectiveness as a concern. Only 9% of participants indicated that they faced no significant barriers to adopting sustainable practices (Figure 2B).

### 3.3 Willingness and Preferred Initiatives for Green Surgery

Figure 2C,D highlight participants' willingness to engage in Green Surgery practices and their preferences for specific sustainability initiatives. The majority expressed a strong willingness to participate, with 63% indicating that they “somewhat would” and 19% affirming that they “definitely would.” Over 80% indicated at least moderate interest in adopting sustainable surgical practices. Only 15% reported that they “slightly would” engage, and just 3% stated that they “definitely would not,” indicating limited resistance to the concept of Green Surgery overall (Figure 2C).

Respondents showed clear support for practical and impactful strategies when presented with options. The most favored initiative was using reusable surgical instruments such as ports, selected by 74%; this was followed by using remanufactured single‐use medical devices (66%) and revising sterilization kits to reduce waste (49%). These preferences suggest a focus on feasible initiatives to lessen surgical procedures' environmental footprint. Additionally, switching to reusable surgical gowns (38%) and participating in sustainability workshops or committees (37%) were also commonly selected, indicating openness to both material‐ and institutional‐level changes (Figure 2D).

## 4 Discussion

The survey findings reveal challenges and opportunities for promoting environmentally sustainable surgical practices within the JSES. While awareness of Green Surgery and specific GHG emissions related to surgical practices is currently limited, there is considerable concern regarding the environmental impact of ORs. Most participants expressed a willingness to adopt sustainable practices, particularly those that are feasible and directly linked to reducing carbon footprints, such as reusable surgical instruments and revised sterilization kits. However, significant barriers remain, including perceived increases in treatment costs, potential disruptions to surgical workflows, and safety concerns regarding patient care. These challenges align with global efforts to integrate sustainability into healthcare practices.

Global warming is rapidly accelerating, with the past two decades witnessing significant increases in global temperatures [8]. This rise has led to more frequent extreme weather events, serious health impacts, and disruptions to agriculture and fisheries, as supported by scientific evidence [8]. As a major contributor to GHG emissions [4, 5], the healthcare sector cannot evade its responsibility to address this global crisis. ORs, in particular, contribute heavily to the sector's carbon footprint owing to their high energy consumption and reliance on disposable instruments [9]. Though beneficial for patients, minimally invasive surgery adds to the environmental burden. For example, a single laparoscopic appendectomy produces 27.4 kg of CO_2_ emissions [10], equivalent to driving a typical gas‐powered car for 183 km. Using four single‐use trocars during a laparoscopic cholecystectomy generates 1.14 kg of CO_2_ equivalents [11], while manufacturing an ultrasonic surgical device produces 3.75 kg of CO_2_ equivalents [12].

Furthermore, robotic surgeries result in 38%–43.5% more GHG and 24%–27.6% more waste than laparoscopic surgeries [13, 14], raising concerns that their environmental impact may outweigh their clinical benefits [15]. Our survey highlights the limited engagement in sustainable practices within ORs in Japan. Despite 63% of respondents expressing moderate concern about the environmental impact of GHG emissions and waste in ORs, only 5% reported a well‐developed understanding of Green Surgery, and 46% indicated that their facilities were “not very engaged” in implementing sustainable practices. Encouragingly, 74% of the participants showed strong support for reusable surgical instruments and 66% favored remanufactured single‐use devices. These preferences align with the findings of Rizan and Bhutta, who demonstrated that adopting environmentally friendly measures, such as hybrid surgical instruments for laparoscopic cholecystectomy, can significantly reduce carbon footprints by 76% compared to single‐use equivalents [16]. Understanding the positive environmental and financial implications of these initiatives could motivate greater efforts toward sustainable surgical practices, addressing both the barriers and opportunities identified in this survey.

Professional societies related to endoscopic surgery have increasingly recognized the importance of addressing the carbon footprint of surgical practices through collaborative initiatives. The Society of American Gastrointestinal and Endoscopic Surgeons (SAGES) and the European Association for Endoscopic Surgery (EAES) established a joint task force on Sustainability in Surgical Practice in 2023 [17]. Their key strategies include measuring and validating the carbon footprint of surgical practices, developing methods to reduce the carbon footprint of ORs, and educating the surgical community on the best practices for sustainability. In alignment with these global efforts, the JSES launched environmental research grants in 2023, selecting two studies for funding, and established a dedicated Working Group on Environmental Issues [7]. This group aims to reduce GHG emissions from endoscopic surgery through initiatives such as research support and environmentally focused programs at annual meetings. The survey among JSES members marks a vital starting point in this movement, providing foundational insights into current awareness, attitudes, and practices. It emphasizes the need to raise awareness regarding Green Surgery and the importance of scientifically evaluating the impact of endoscopic surgery on global warming. While many healthcare professionals focus on sustainability's environmental and operational aspects, its impact on public health should not be overlooked. Our findings indicate that many participants recognized the health effects of climate change, suggesting that greater awareness of these effects may help promote interest in sustainable surgical practices. Additionally, it is essential to propose practical and evidence‐based strategies adapted to the context of Japan's healthcare system, through activities such as academic initiatives and guideline development.

We present seven strategies that translate the concept of Green Surgery into actionable steps as follows:

1. Understanding Green Surgery: The first step for surgeons and healthcare staff is acknowledging the importance of environmentally friendly surgical practices.
2. Minimizing medical waste: This remains a critical intervention in promoting sustainability.
3. Rethinking anesthesia: Anesthetic gases considerably contribute to GHG emissions [18, 19]. Selecting low‐impact alternatives and optimizing techniques can mitigate this environmental burden.
4. Avoiding unnecessary openings of sterile supplies: A substantial portion of OR waste comes from open but unused supplies [20, 21]. Opening only what is necessary is an effective measure that can be implemented immediately.
5. Optimizing surgical procedures: Optimizing surgical procedures to reduce human and material resources is essential for achieving Green Surgery.
6. Switching to reusable products: Prioritizing reusable surgical instruments and supplies can considerably reduce GHG emissions and waste while maintaining patient safety [14, 22, 23, 24].
7. Encouraging the development of sustainable products: Our society will advocate for medical device manufacturers to develop products that minimize environmental impacts, including reducing CO_2_ emissions and mitigating GHG emissions.

These strategies should be gradually implemented with careful attention to maintaining infection control and patient safety. By addressing these critical considerations, adopting environmentally sustainable practices can support both clinical priorities and green surgical goals. For those seeking further guidance, a comprehensive resource titled “Green Surgery: Reducing the environmental impact of surgical care”, developed collaboratively by UK‐based organizations including Brighton and Sussex Medical School, provides detailed recommendations supported by evidence and case studies from clinical practice in the UK [25].

This study has several limitations. First, the respondents represented only approximately 10% of JSES members, which may not fully capture the opinions of the entire membership. This relatively low participation rate could indicate limited awareness of and interest in environmental sustainability within the Japanese surgical community. Those who participated are likely more environmentally conscious, potentially introducing selection bias. This may lead to an overestimation of overall concern regarding environmental issues and the willingness to adopt Green Surgery practices among JSES members. Second, the reliance on self‐reported data may not accurately reflect actual behaviors or the levels of implementation of sustainable practices. Finally, while the study provides insights into general trends and perceptions, it lacks a specific analysis of institutional policies or practical implementation strategies for driving systemic changes. Addressing these limitations requires further research and comprehensive evaluation. Despite these limitations, this survey is an important foundation for fostering discussions on the environmental impacts of surgical practices in Japan.

In conclusion, this survey highlighted the current awareness, attitudes, and practices related to environmentally sustainable surgical practices among JSES members, revealing both significant barriers and opportunities for progress. While challenges and differing opinions exist, fostering constructive and forward‐looking discussions is critical for advancing Green Surgery. Building on these findings, the JSES can lead in integrating sustainable practices into Japan's healthcare system, thereby contributing to global sustainability goals.

## Author Contributions

**Naoki Ikenaga:** writing – review and editing. **Masafumi Nakamura:** conceptualization. **Mizuki Hirasawa:** investigation. **Takeshi Naitoh:** validation. **Yoshiharu Sakai:** methodology. **Tomonori Habuchi:** supervision. **Yuko Kitagawa:** supervision. All authors critically reviewed and approved the manuscript.

## Conflicts of Interest

Dr. Takeshi Naitoh, Dr. Yoshiharu Sakai, and Dr. Tomonori Habuchi are the Editorial Board members of Asian Journal of Endoscopic Surgery and a co‐authors of this article. To minimize bias, they were excluded from all editorial decision‐making related to the acceptance of this article for publication.

## Supporting information


**File S1.** Survey items and response options.

**Table S1.** Demographics of participants.

## Data Availability

The authors have nothing to report.
