# Supplementary material for: Green Surgery Awareness and Challenges: A Survey Among Members of the Japan Society for Endoscopic Surgery
Source: Asian J Endosc Surg. 2025 May 29;18(1):e70087. doi: 10.1111/ases.70087 (PMC12122130; doi:10.1111/ases.70087)
Supplement: Supplementary file 1 — File S1. Survey items and response options. [file ASES-18-e70087-s002.docx]

**Supplementary file 1. Survey Items and Response Options**

**Section 1: Demographics**

1. What is your age? 20s / 30s / 40s / 50s / 60+

2. What is your gender? Male / Female / Neither one / No response

3. How many years of medical experience have you?

1. Less than 10 years
2. 10–19 years
3. 20–29 years
4. 30–39 years
5. 40+ years

4. What is your job title?

1. Director
2. Deputy Director
3. Department Head
4. Staff
5. Resident
6. Other

5. What is the type of your workplace?

1. University Hospital
2. Public Hospital (Teaching)
3. Public Hospital (Non-Teaching)
4. Private Hospital (Teaching)
5. Private Hospital (Nonteaching)
6. Clinic
7. Other

6. How many beds are there in your workplace?

1. Fewer than 25
2. 25–100
3. 100–400
4. 400–800
5. More than 800

7. What is your specialty?

1. Gastroenterology and General Surgery
2. Thoracic Surgery
3. Gynecology
4. Urology
5. Pediatric Surgery
6. Orthopedic Surgery
7. Other

**Section 2: Current Knowledge**

8. How well do you understand “Green Surgery” (environmentally friendly surgical practices)?

1. Well
2. Moderately well
3. Slightly well
4. Not at all

9. How concerned are you about the amount of greenhouse gases and waste generated in operating rooms?

1. Very concerned
2. Moderately concerned
3. Slightly concerned
4. Not concerned at all

10. Do you think that greenhouse gas emissions and waste generated in operating rooms impact the environment?

1. Significantly impact the environment
2. Moderately impact the environment
3. Slightly impact the environment
4. Do not impact the environment

11. Do you think that climate change affects patients’ health?

1. Significantly impact the environment
2. Moderately impact the environment
3. Slightly impact the environment
4. Do not impact the environment

12. How well do you understand greenhouse gas emissions are associated with the entire lifecycle of surgical instruments (e.g., ultrasonic coagulation cutting devices), including raw material extraction, manufacturing, distribution, use, and disposal or recycling?

1. Fully aware
2. Moderately aware
3. Slightly aware
4. Not aware at all

13. How familiar are you with the greenhouse gas emissions generated during surgical procedures (e.g., laparoscopic cholecystectomy)?

1. Fully aware
2. Moderately aware
3. Slightly aware
4. Not aware at all

**Section 3: Perspectives on future initiatives**

14. What are your concerns regarding promoting Green Surgery (an environmentally friendly surgical practice)? **(■ Multiple selections allowed)**

1. Increased treatment costs
2. Decreased treatment effectiveness
3. Decreased patient safety
4. Changes in surgical procedures or the inability to perform certain surgeries
5. Increased working hours
6. None

15. Do you think that Green Surgery (an environmentally friendly surgical practice) can impact climate change?

1. I think it has a significant impact
2. I think it has a moderate impact
3. I think it has little impact
4. I think it has no impact
5. I don’t know

16. Is your facility actively engaged in environmentally friendly operating room management?

1. Fully engaged
2. Somewhat engaged
3. Not very engaged
4. Not engaged at all
5. Do not know

17. Would you like to engage in Green Surgery practices (environmentally friendly)?

1. Definitely would
2. Somewhat would
3. Slightly would
4. Definitely would not

18. How much discretion do you have in selecting surgical instruments in the operating room?

1. I have significant discretion
2. I have some discretion
3. I have little discretion
4. I have no discretion

19. What actions would you like to implement in the future to promote Green Surgery (environmentally friendly surgical practices)? **(■ Multiple selections allowed)**

1. Switch to reusable surgical gowns
2. Use reusable surgical instruments (e.g., ports)
3. Use remanufactured single-use medical devices
4. Propose eco-friendly anesthetic methods to anesthesiologists
5. Revise sterilization kits
6. Establish sustainability committees and/or participate in such workshops
7. None

20. How would you like to learn about Green Surgery (environmentally friendly surgical practice)?

**(■ Multiple selections allowed)**

1. In-person lectures
2. Online lectures
3. Group workshops
4. Watching educational videos
5. Other (free text)
6. I do not want to learn

21. What role do you expect the JSES to play in promoting surgical sustainability?

Free text
