# Supplementary material for: Green Surgery Awareness and Challenges: A Survey Among Members of the Japan Society for Endoscopic Surgery
Source: Asian J Endosc Surg. 2025 May 29;18(1):e70087. doi: 10.1111/ases.70087 (PMC12122130; doi:10.1111/ases.70087)
Supplement: Supplementary file 2 — Table S1. Demographics of participants. [file ASES-18-e70087-s001.docx]

**Supplementary Table 1 |** Demographics of participants.

| Age | 20s  30s  40s  50s  60+ | 0.6%  12%  35%  38%  15% |
| --- | --- | --- |
| Gender | Male  Female  Neither one  No response | 91%  9%  0.1%  0.4% |
| Medical experience | Less than 10 years  10–19 years  20–29 years  30–39 years  40+ years | 5%  25%  41%  24%  5% |
| Job title | Director  Deputy Director  Department Head  Staff  Resident  Others | 5%  9%  33%  45%  3%  5% |
| Workplace type | University Hospital  Public Hospital (Teaching)  Public Hospital (Non-Teaching)  Private Hospital (Teaching)  Private Hospital (Non-Teaching)  Clinic  Other | 37%  28%  6%  18%  8%  2%  1% |
| Specialty | Gastroenterology and General Surgery  Thoracic Surgery  Gynecology  Urology  Pediatric Surgery  Orthopedic Surgery  Others | 69%  10%  7%  8%  4%  0.4%  1% |
